# HIV pre‐exposure prophylaxis for female sex workers: ensuring women’s family planning needs are not left behind

**DOI:** 10.1002/jia2.25442

**Published:** 2020-02-17

**Authors:** Anna L Bowring, Frances H Ampt, Sheree Schwartz, Mark A Stoové, Stanley Luchters, Stefan Baral, Margaret Hellard

**Affiliations:** ^1^ Department of Epidemiology Johns Hopkins School of Public Health Baltimore MD USA; ^2^ Burnet Institute Melbourne Victoria Australia; ^3^ Department of Epidemiology and Preventive Medicine Monash University Melbourne Victoria Australia; ^4^ Department of Population Health Aga Khan University Nairobi Kenya; ^5^ International Centre for Reproductive Health Department of Public Health and Primary Care Ghent University Ghent Belgium; ^6^ Department of Infectious Diseases The Alfred Hospital Melbourne Victoria Australia; ^7^ Doherty Institute and Melbourne School of Population and Global Health University of Melbourne Melbourne Victoria Australia

**Keywords:** sex workers, Pre‐Exposure Prophylaxis (PrEP), contraception, unplanned pregnancy, HIV infections

## Abstract

**Introduction:**

Female sex workers (FSWs) experience overlapping burdens of HIV, sexually transmitted infections and unintended pregnancy. Pre‐exposure prophylaxis (PrEP) is highly efficacious for HIV prevention. It represents a promising strategy to reduce HIV acquisition risks among FSWs specifically given complex social and structural factors that challenge consistent condom use. However, the potential impact on unintended pregnancy has garnered little attention. We discuss the potential concerns and opportunities for PrEP to positively or negatively impact the sexual and reproductive health and rights (SRHR) of FSWs.

**Discussion:**

FSWs have high unmet need for effective contraception and unintended pregnancy is common in low‐ and middle‐income countries. Unintended pregnancy can have enduring health and social effects for FSWs, including consequences of unsafe abortion and financial impacts affecting subsequent risk‐taking. It is possible that PrEP could negatively impact condom and other contraceptive use among FSWs due to condom substitution, normalization, external pressures or PrEP provision by single‐focus services. There are limited empirical data available to assess the impact of PrEP on pregnancy rates in real‐life settings. However, pregnancy rates are relatively high in PrEP trials and modelling suggests a potential two‐fold increase in condomless sex among FSWs on PrEP, which, given low use of non‐barrier contraceptive methods, would increase rates of unintended pregnancy. Opportunities for integrating family planning with PrEP and HIV services may circumvent these concerns and support improved SRHR. Synergies between PrEP and family planning could promote uptake and maintenance for both interventions. Integrating family planning into FSW‐focused community‐based HIV services is likely to be the most effective model for improving access to non‐barrier contraception among FSWs. However, barriers to integration, such as provider skills and training and funding mechanisms, need to be addressed.

**Conclusions:**

As PrEP is scaled up among FSWs, there is growing impetus to consider integrating family planning services with PrEP delivery in order to better meet the diverse SRHR needs of FSWs and to prevent unintended consequences. Programme monitoring combined with research can close data gaps and mobilize adequate resources to deliver comprehensive SRHR services respectful of all women’s rights.

## 1 Introduction

Female sex workers (FSWs) in low‐ and middle‐income countries experience overlapping burdens of high HIV prevalence alongside high rates of sexually transmitted infections (STIs) and unintended pregnancy 1, 2. Despite these coexisting reproductive health needs, in many settings family planning is poorly integrated into sexual and reproductive health and rights (SRHR) services for FSWs, which often maintain a narrow focus on HIV and STI prevention and treatment 3, 4, 5. Dual protection, denoting consistent condom use together with another effective contraceptive method, is currently the recommended approach to minimize risk of HIV/STIs and unintended pregnancy. However, the use of effective non‐barrier contraception methods among FSWs is sub‐optimal, resulting in high rates of unintended pregnancy 2. Similarly, FSWs’ condom use, even when the primary method of pregnancy prevention, is often inconsistent, particularly with non‐paying partners 6, 7, 8.

Pre‐exposure prophylaxis (PrEP) is a relatively new intervention being offered to FSWs and other populations with high risk of HIV acquisition. Although the potential benefits and acceptability of PrEP among FSWs have been previously established 9, 10, 11, 12, 13, 14, specific consideration of the implications of PrEP for family planning in this population is lacking.

Depending on approaches to scale‐up, there is the possibility that PrEP programming could increase rates of unintended pregnancy among FSWs. Risk compensation, normalization of PrEP use and external pressures for condomless sex could impact on condom use, while PrEP provision within single‐focus services that insufficiently cater to family planning needs could impact use of other contraceptive methods. Drawing from our experience in working with national PrEP implementation programmes, the purpose of this commentary is to elaborate on the context in which PrEP is being implemented with FSWs and the potential concerns and opportunities for PrEP to impact unintended pregnancy and other aspects of SRHR.

## 2 Discussion

### 2.1 Context and interactions between PrEP and SRHR

#### 2.1.1 *Benefits of PrEP for* FSWs

PrEP offers personal control over HIV, and is therefore a prevention option of particular benefit to FSWs because it can help counter pervasive social and structural factors that limit FSWs’ capacity to consistently use condoms to prevent HIV, including gender norms, criminalization, intersecting stigmas and sexual and physical violence 9, 15. In particular, PrEP can mitigate risks of HIV acquisition associated with condom non‐use or condom breakage 15, 16, including protecting against HIV acquisition with non‐paying, emotional partners 17, 18. PrEP also provides an effective way to reduce risk of HIV for FSWs and their partners who wish to conceive 19, 20. There is strong evidence that PrEP is efficacious in preventing HIV infection among women when used consistently and as directed 21.

#### 2.1.2 *Contraceptive use and unintended pregnancy among* FSWs

Preventing pregnancy may be the greater motivator for FSWs to use condoms compared to HIV prevention 13, 22. FSWs across low‐ and middle‐income countries experience high rates of unintended pregnancy: in cohort studies without SRHR interventions, the pooled incidence rate for unintended pregnancies among FSWs is 27.1 per 100 person‐years (95% CI = 24.4 to 29.8) 2. Use of effective non‐barrier contraceptive methods among FSWs is below 40% in numerous settings across sub‐Saharan Africa 7, 23, 24, 25 with few FSWs in the region using long‐acting reversible contraceptives (LARCs) – intrauterine devices and implants – despite their superior effectiveness 2, 26. User‐dependent methods such as injections and pills are often used inconsistently or incorrectly 27, 28. Injections are the most commonly used and often the only available non‐barrier method in this population 13, 24, 25, 29 but are reported to have acceptability concerns among FSWs due to side effects which interfere with their ability to work 13, 30. Additional barriers to access and uptake of contraception among FSWs include stigma or refusal of service due to sex work, young age or marital status, limited availability or choice of contraceptive methods, lack of health workers trained in LARC provision and limited knowledge of contraceptive methods 5, 31, 32, 33.

#### 2.1.3 Risk compensation

The potential for individuals on PrEP to reduce their use of condoms due to negation of HIV risk is a commonly voiced concern 34. Nonetheless, acceptability studies suggest that FSWs consider PrEP a favourable user‐controlled back‐up option for when condoms fail or cannot be used, rather than an alternative 12, 13, 35. Based on a recent meta‐analysis, individuals starting PrEP have a high burden of STIs 36. Furthermore, there is emerging evidence from open‐label and demonstration studies and implementation sites among gay, bisexual and other men who have sex with men (MSM) that condom use decreases among some men on PrEP, particularly among those already engaging in condomless sex or other high‐risk behaviours 37, 38, 39, 40, 41. However, modelling among MSM suggest that if PrEP is accompanied by regular STI services, increased detection and treatment may mitigate increases in STI transmission 42, 43. While there is no current evidence among FSWs of decreasing condom use or other behaviours increasing vulnerability to STIs and unintended pregnancy 44, 45, 46, implementation data are too limited to discount this possibility. It is possible that risk compensation will increase over time as PrEP becomes more normalized 39. Importantly, price premiums for condomless sex are already reported by FSWs in many settings 47, 48, 49, and as community knowledge of PrEP increases, so too may client demand and pressure for condomless sex 10, 50, 51. Of note, modelling in South Africa has suggested that condomless sex may increase two‐fold among FSWs on PrEP by lowering negotiating power and increasing coercion by clients 51. While undocumented, it is also possible that individuals in a position of disparate power or economic control over FSWs may coerce FSWs to use PrEP whether or not they consider it as the most suitable HIV prevention option for their circumstances.

#### 2.1.4 Impacts of unintended pregnancy

Most concerns raised around the potential implications of condomless sex focus on STIs – perhaps given the predominance of PrEP studies involving MSM in the literature. Although less frequently considered, mistimed or unwanted pregnancies are a potential adverse outcome of PrEP use that may have enduring social and health consequences. Among FSWs, unintended pregnancy is commonplace and is often followed by unsafe abortion 33. Restrictive abortion policies throughout most of Africa result in three‐quarters of abortions in the region being unsafe, contributing to 10% of maternal deaths 52, 53, 54. Furthermore, restrictive donor policies prevent programmatic support for identifying safer termination options for women, as referrals or counselling around pregnancy options are prohibited under the renewed United States’ global gag rule 55.

In addition to the immediate health risks of unintended pregnancy due to unsafe abortion and high maternal mortality in areas most affected by HIV 56, pregnancy and caring for children has major economic implications for FSWs that can lead to subsequent risk. While motivations for selling sex are complex and multifactorial 57, one reason often reported is the need for financial independence among women with children who are not financially supported by a partner 57, 58, 59. Unintended motherhood may increase economic dependency on sex work and pressure women to take more clients, accept condomless sex for more money, or serve as a barrier to leaving sex work, thus increasing or prolonging risk for HIV and other STIs 59, 60, 61, 62. A study in India found that FSWs with more children were more likely to report inconsistent condom use and accept more money for condomless sex 60. In Tanzania, sex workers reported increasing sexual risk behaviours to help fund their children’s school fees 63. Pregnancy and childcare may also lead to women taking unplanned or unwanted breaks from sex work, thus adding to household insecurity 64. Conversely, for some FSWs, responsibility to care for children may be a motivator to use PrEP in order to stay healthy and maintain their earning potential 12, 35, 63.

#### 2.1.5 Evidence from PrEP studies

Evidence of the impact of PrEP on pregnancy rates among all women is scant. The FEM‐PrEP randomized controlled trial (RCT) did not show a significant impact of PrEP compared to placebo on unintended pregnancy among women 65, 66. As use of contraception was a condition of enrolment and supplied to participants, this result suggests that PrEP does not reduce the efficacy of hormonal contraception 67, but it cannot be used to predict the influence of PrEP on behaviour in real‐life settings. Pregnancy incidence has not been reported in contexts that would give a better indication of the real‐world effect of PrEP, specifically among FSWs, such as demonstration studies, open‐label comparisons of women using versus not using PrEP, or monitoring of larger scale PrEP programmes 44, 45. Nonetheless, pregnancies in PrEP trials were relatively common among both PrEP and control groups with rates comparable to general rates in developing countries 68, even when baseline criteria had stipulated contraceptive use and no current pregnancy intentions and provided access to at least shorter acting contraceptive methods: the overall pregnancy incidence was 10 per 100 person‐years in both the Partners PrEP and FEM‐PrEP studies 65, 69, and approximately 8 per 100 person‐years in the FACTS 001 tenofovir gel and VOICE trials 70, 71. High rates of unintended pregnancies have also been reported in older PrEP trials 22 and trials of other HIV prevention technologies 72, 73, often in the context of free provision of contraceptives 69, 73.

#### 2.1.6 Contraceptive method mix for PrEP users

Choice of contraceptive is important, and in FEM‐PrEP, the pregnancy rate was much higher among those using oral contraceptive pills (incidence rate 31.7 per 100 person years overall) compared to injectables (incidence rate 1.6 per 100 person years) or long‐acting or permanent methods (none reported) 65; similar findings were reported from VOICE 74. This highlights the need to promote and supply longer acting methods and deliver high‐quality contraceptive counselling 72 in the context of PrEP provision. Importantly, many PrEP trials provided oral and injectable contraceptives onsite but referral for LARCs 65, 74, which may have been a deterrent to accessing more effective contraception. Trials have also indicated higher pregnancy incidence among new versus established contraceptive users, especially among oral contraceptive users, emphasizing the need for counselling support for new users 65, 74. Recent findings from the ECHO contraceptive trial showed no difference in HIV risk between three commonly available contraceptive methods in Sub‐Saharan Africa 75. However, just as high rates of pregnancy have been observed in PrEP trials, high rates of HIV acquisition were observed in the ECHO trial across all contraceptive arms, despite the integration of HIV risk reduction counselling, condom provision, HIV and STI testing and eventually PrEP, when it became locally available. Considering there are unmet HIV and family planning needs in the context of well‐resourced and closely monitored HIV and contraceptive trials, the scale‐up of these services requires effective service integration to meet the breadth of SRHR needs in targeted populations.

### 2.2 Opportunities and considerations for family planning and PrEP integration

There are opportunities to meet the HIV prevention needs of many FSWs while minimizing additional unintended pregnancy risk through the integration of PrEP and family planning services. Such service integration would also enhance opportunities for STI prevention and treatment and could promote more efficacious approaches such as LARCs and multipurpose prevention technologies for combined HIV prevention and contraception.

#### 2.2.1 Synergies between PrEP and family planning services

PrEP and family planning services for FSWs are likely to be complementary. PrEP adherence and maintenance among FSWs with sustained high‐risk behaviours remains a major challenge, with studies consistently demonstrating low PrEP continuation among FSWs even at one month 44, 45, 76, 77. Provision of complementary services such as STI testing has been associated with longer maintenance of PrEP 77, 78. Integration of family planning may also facilitate PrEP uptake and continuation, as women seeking contraception or wanting to conceive may be ideal PrEP candidates. Regular monitoring of individuals on PrEP also provides opportunities for contraception renewal and vice versa. Building on these synergies between family planning and PrEP may reduce the administrative burden to both service providers and clients and promotes more cost‐effective service provision 79, 80. Shared social, economic and structural factors underlying vulnerability to both unintended pregnancy and HIV acquisition, such as violence, substance use and financial insecurity, may also affect uptake of PrEP and family planning 81, 82. Programmes which address these factors may support uptake and adherence to PrEP, contraception and condoms alike 83.

#### 2.2.2 Models of PrEP and family planning service integration

Current programmes providing PrEP to FSWs are predominantly implemented through FSW‐focused services, which include drop‐in centres and clinics led by community‐based organizations 84. Acceptability studies indicate that these focused services are often the preferred way for FSWs to access healthcare due to staff friendliness, lower cost, shorter waiting times, privacy, proximity to places of work and greater quality of care 8, 81, 85, 86. There are several advantages of building upon this model for integrated PrEP and family planning delivery. FSW‐focused services commonly utilize peer‐based prevention and community mobilization programmes and may provide opportunities to address syndemic vulnerabilities as well as informational, structural and social barriers to the use of PrEP and contraception, including condoms 4, 81, 86. Community mobilization approaches promoting social cohesion, leadership and empowerment have been empirically associated with reductions in HIV and STI acquisition and increased treatment adherence 83, 87. With appropriate training, leadership and buy‐in, community health workers and peer educators can play an important role in supporting PrEP and reproductive health provision. Community‐supported models of care are already important for delivery of antiretroviral therapy in numerous settings 88. Providing options for PrEP and family planning through mobile service delivery may extend service access and acceptability among FSW 13, 89. The potential of FSW‐focused services to provide comprehensive SRHR care can be further realized by the addition of pregnancy testing, STI management, gender‐based violence services and cervical cancer screening 3.

Alternative models for the integration of family planning and HIV services have focused on antenatal or family planning services 90, 91. There are emerging data supporting the feasibility of integrating PrEP into family planning clinics targeting adolescent girls and young women 92, 93, although these may have limited utility and acceptability for FSWs due to potential stigmatization. FSWs and women who are pregnant outside of marriage are often stigmatized, shamed, denied care in public clinics, or are provided limited contraceptive choice based on assumptions about their needs and behaviours 5, 94, 95, 96, 97. However, this model of integration may still be valuable in contexts where FSWs are already accessing services in public or private antenatal or family planning facilities 85. Lessons learned from these studies highlight that PrEP and family planning integration does not only confer synchronized commodity delivery in a common space; there is also a need to integrate tools for screening, monitoring and evaluation, coordinate demand generation activities and integrate training 92.

Family planning entails not only provision of contraception, but also non‐judgemental and non‐coercive discussions around method options, fertility desires and safer conception counselling. Based on learnings from SRHR, regardless of integration model, providing multiple contraceptive options 98 and counselling and provision of LARCs over other non‐barrier methods where possible 31, 32 may optimize contraceptive uptake and acceptability among FSW. Counselling on contraceptive options should consider individuals’ existing contraceptive use as well as current needs, as oral contraceptives may be less effective among new users in particular 65.

#### 2.2.3 Barriers to integration

There are several barriers to realizing integrated PrEP and family planning services. Common reliance on donor‐driven funding schemes favour vertical, disease‐specific programming, thus limiting the availability of sufficient funding for adequate family planning and STI services within HIV programmes 99, 100. This is exacerbated by inadequate mobilization of domestic funding for HIV prevention programmes, particularly among key populations 101. The management and procurement of PrEP and family planning commodities through different funding and sources may pose additional challenges to integrated delivery. Furthermore, the community‐based nature of many services reaching FSWs and other key populations 102 can limit the availability of staff with suitable qualifications and training to deliver LARCs 26, 79, 103. Finally, there is currently a lack of policy guidance to support implementers to integrate family planning into their programmes, with most national PrEP policies and guidelines making fleeting references to including contraceptive counselling within a combination package of prevention services 104, 105, 106. Means to overcome these barriers without relying on referrals to other services include task‐shifting contraceptive administration to lower‐cadre healthcare workers 107, 108 and collaborations between family planning and HIV services to hold recurrent “family planning days” at FSW‐friendly community services which are attended by trained personnel for LARC administration. Enabling policies, clear implementation guidelines and adequate systems for commodity supply and distribution may support task‐shifting and service integration 109. “Diagonal” models of care which leverage the international vertical funding for PrEP and other HIV prevention programmes 110, and incorporate it into existing SRHR services focused on FSWs, may also facilitate more effective integration. Intersectional stigmas remain major barriers to PrEP uptake among key populations in many settings 111, 112. Interventions aimed at mitigating PrEP‐related stigmas in the community, as well as during PrEP provision and family planning services, through careful marketing and peer‐led, confidential and non‐judgemental services may potentiate PrEP uptake and retention and integration with reproductive health services 13, 81, 89.

STI management is already commonly integrated in HIV programmes for FSW 3. However, many programmes among FSWs in resource‐limited settings currently rely on syndromic management of STIs, and given that the majority of STIs are asymptomatic, this approach is likely to result in untreated STIs and ongoing transmission 113, 114. To uphold improvements in SRHR through integrated PrEP delivery, there is demonstrated need to invest in improvements for STI services, including low‐cost point‐of‐care STI tests where possible 115, vaccination for human papilloma virus and viral hepatitis, and other modalities for STI management 116.

#### 2.2.4 Multipurpose devices for prevention of HIV and pregnancy

Combined delivery of PrEP and hormonal contraception could be a convenient and efficient means of simultaneously preventing HIV and unintended pregnancy. Multipurpose prevention technologies for modern contraception and HIV are in early development stages, including intravaginal ring and gel preparations. A dual‐purpose intravaginal ring delivering dapivirine and levonorgestrel has undergone Phase I trials through the MTN‐030/IPM‐041 study in the US and demonstrated high tolerance and safety 117. However, similar technologies for PrEP delivery demonstrated only moderate effectiveness, reducing HIV incidence by 27% 118 and 31% 119 in Phase III trials. Long‐acting multipurpose technologies have the potential to ease barriers to adherence, and intravaginal rings have the advantage of providing direct delivery to the predominant site of viral transmission among most women. However, one study among FSWs in Tanzania found lower acceptability of the intravaginal ring compared to other PrEP modalities 120. Furthermore, while long‐acting injectable multipurpose technologies may have greater acceptability 120, single‐purpose injectable PrEP is still under Phase III trial for PrEP efficacy and thus more immediate interventions are needed 121. In the near‐term, this might include options for co‐packaging PrEP with oral contraceptives. However, this should still be considered in light of the limitations of oral contraceptives, in particular lower effectiveness compared to LARCs and pill burden 65. Finally, despite synergies between HIV and unintended pregnancy risks, HIV risks and pregnancy intentions are each dynamic and temporal needs may not always synchronize. The ease of transitioning to single‐purpose prevention modalities due to change in needs or fertility desires must also be considered.

## 3 Conclusions

Given the significant social and health impacts of unintended pregnancy for FSWs, there is impetus to consider family planning as an integral component of PrEP programming. Surprisingly, the potential for unintended pregnancy in the provision of PrEP use has garnered limited attention to date despite well‐documented concerns around condom migration and STI risk. Bi‐directional integration of PrEP and family planning could have a broad positive impact on FSWs across low‐ and middle‐income countries. In view of the complex social and structural influences on condom use, settings with low use of non‐barrier methods pose a major concern in terms of unintended pregnancy risk and should be prioritized for integrated delivery of PrEP and family planning. As PrEP is scaled up among FSWs, it remains important to measure the unintended consequences of PrEP as well as its benefits. Systematic collection and reporting of implementation data pertaining to PrEP, family planning and related outcomes will support the optimization of integration strategies to meet the multifaceted needs of FSWs. Furthermore, dedicated research is needed to test the hypotheses that integrating contraception with PrEP delivery improves PrEP continuation and decreases unintended pregnancy and to compare models of PrEP and family planning integration. These data can be used by researchers and programmatic implementers alike to mobilize adequate resources to deliver comprehensive SRH services respectful of all women’s SRH needs and reproductive rights.

## Competing interest

The authors have no competing interest to declare.

## Authors’ contributions

MH proposed the initial concept. ALB took the lead role in writing the manuscript, reviewing the literature and developing the initial framework. FHA, SS, SL, MS, SB and MH provided valuable feedback into the content and structure of the manuscript. All authors provided intellectual input on the contents and perspective of the commentary.
